# Demographic Disparities in Clinical Trials of Xanomeline–Trospium for Schizophrenia: A Cross-Sectional Analysis of Data from ClinicalTrials.gov

**DOI:** 10.1192/j.eurpsy.2026.10989

**Published:** 2026-07-31

**Authors:** A. Kwaśny, D. Świeczkowski, A. Włodarczyk, A. Wilkowska, J. Słupski, W. J. Cubała

**Affiliations:** 1 Department of Psychiatry; 2 Department of Toxicology, Medical University of Gdańsk, Gdańsk; 3 Department of Clinical Research, LUXMED Group, Warsaw, Poland

## Abstract

**Introduction:**

Xanomeline, a muscarinic receptor agonist with preferential affinity for the M₁ and M₄ subtypes, has demonstrated antipsychotic efficacy, thereby revitalizing interest in muscarinic receptor–targeted pharmacotherapy for schizophrenia. To mitigate xanomeline-associated peripheral cholinergic adverse effects, it has been co-formulated with trospium chloride, a peripherally restricted muscarinic receptor antagonist. On 26 September 2024, the United States Food and Drug Administration (FDA) approved the oral co-formulation of xanomeline and trospium chloride for the treatment of schizophrenia in adults (Fabiano et al. ECNP 2025; 92, 62-73). Despite substantial advances in neuropsychopharmacology, the persistent underrepresentation of diverse demographic groups in clinical trials remains a widely acknowledged barrier to both scientific understanding and the equitable translation of research findings into clinical practice.

**Objectives:**

The present cross-sectional analysis seeks to characterize the demographic composition of participants enrolled in clinical trials evaluating xanomeline–trospium, drawing upon publicly accessible data from ClinicalTrials.gov.

**Methods:**

A comprehensive search of ClinicalTrials.gov was performed on the 26 April 2025 (see Figure 1). Trials were included if they met the following eligibility criteria: (1) interventional study design; (2) availability of published results on ClinicalTrials.gov; (3) a primary investigational focus on schizophrenia; and (4) classification as a phase 2, phase 3, or phase 4 clinical trial. For each eligible trial, data were extracted on the National Clinical Trial (NCT) identifier, total sample size, study site location, and participant demographic characteristics, including age, sex, race, and ethnicity.

**Results:**

Five clinical trials conducted in the United States and Ukraine (n = 842) met the inclusion criteria. Black participants comprised 68.76% of the pooled sample, White participants 29.45%, and Asian participants 1.07%. Representation from other racial groups, including American Indian or Alaska Native, and Native Hawaiian or Other Pacific Islander, was negligible. At least 8.3% of participants identified as Hispanic or Latino, whereas no fewer than 70.43% were classified as Not Hispanic or Latino; ethnicity data were not reported for trial NCT03697252. Women accounted for 24.7% of participants, and the mean age across trials ranged from 42.5 to 45.9 years (see Table 1). Some trials did not provide complete demographic information.

**Image 1:**
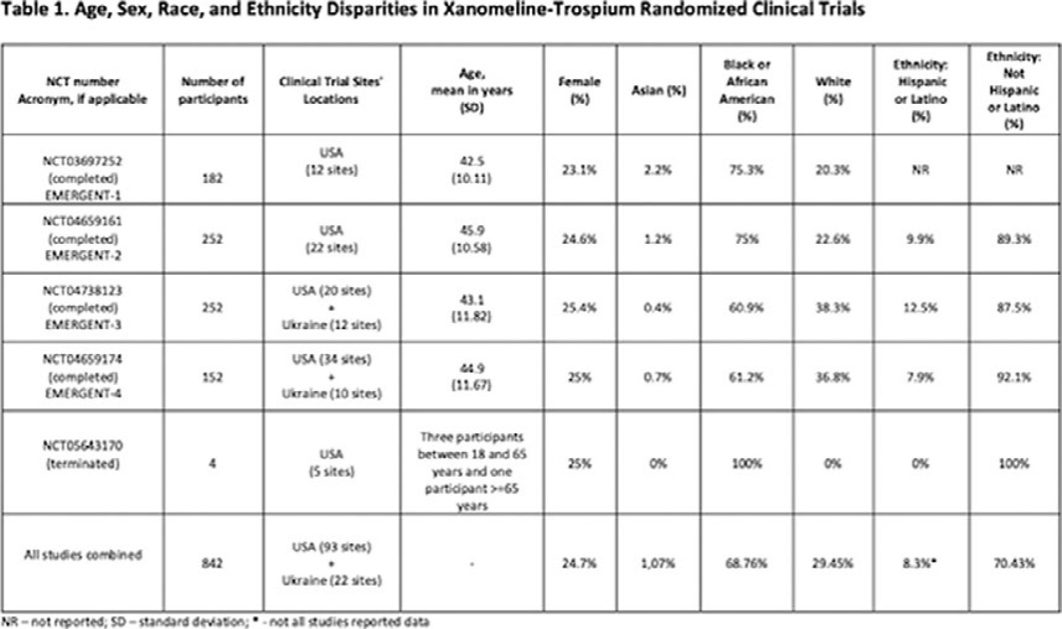
Image 1: Long description.

**Image 2:**
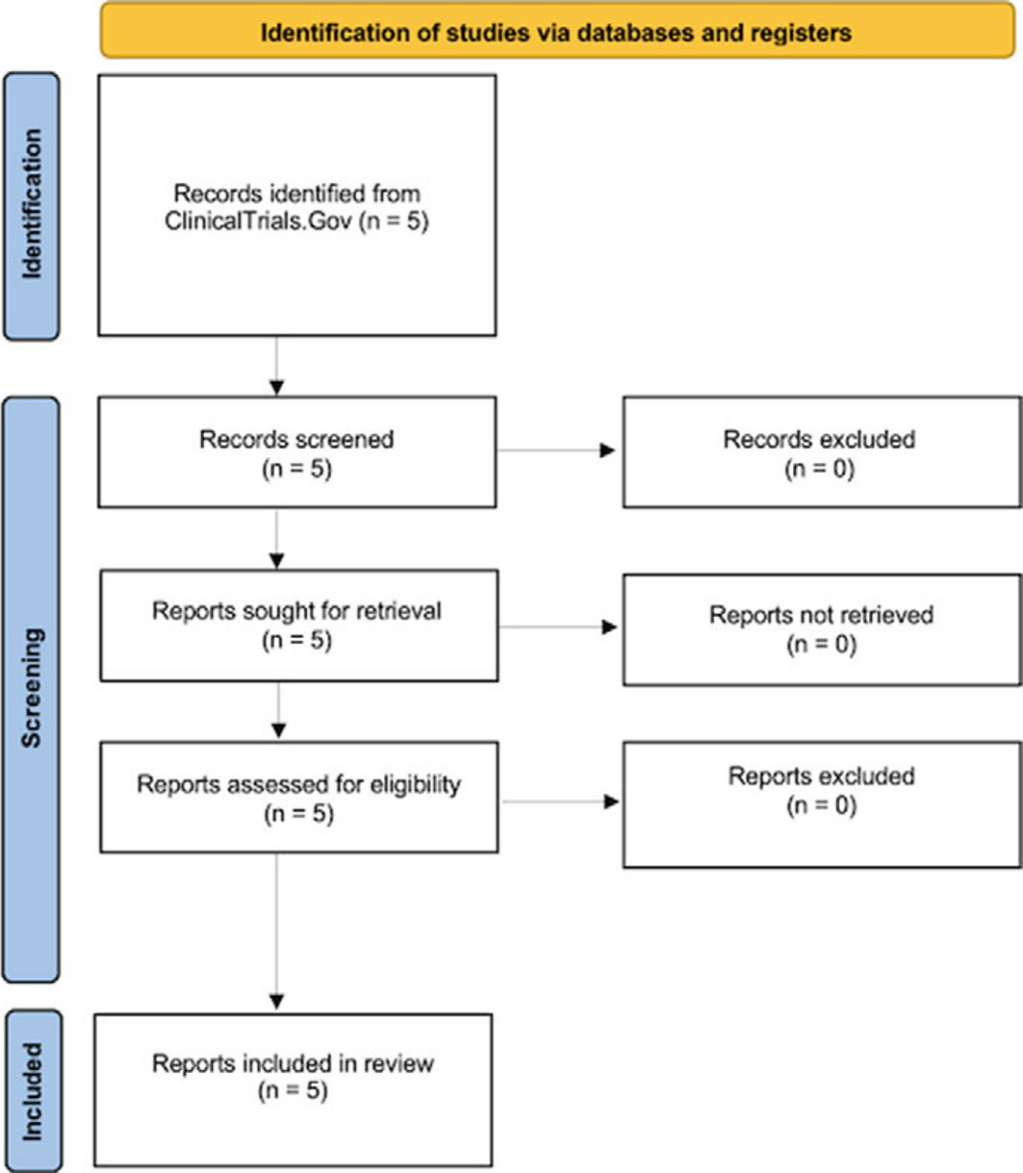
Image 2: Long description.

**Conclusions:**

The findings reveal substantial demographic disparities and underscore the imperative for more inclusive and representative recruitment strategies in future clinical trials investigating schizophrenia.

**Disclosure of Interest:**

A. Kwaśny Grant / Research support from: Beckley Psytech, Compass Pathways, GH Research, MSD., D. Świeczkowski: None Declared, A. Włodarczyk Grant / Research support from: Karuna Therapeutics., A. Wilkowska Grant / Research support from: Angelini, Biogen, Eli Lilly and Company, Janssen-Cilag, Lundbeck, Polpharma, Sanofi, and Valeant., J. Słupski Grant / Research support from: Actavis, Eli Lilly, Minerva Neurosciences, Sunovion Pharmaceuticals, KCR, Janssen, Otsuka, Apodemus, Cortexyme, MSD, Novartis, Ketabon HMNC Brain Health, Acadia and Karuna Therapeutics., W. Cubała Grant / Research support from: Grants: Acadia, Alkermes, Allergan, Angelini, Auspex Pharmaceuticals, Beckley Psytech, BMS, Celon, Cephalon, Compass Pathways, Cortexyme, Ferrier, Forest Laboratories, GedeonRichter, GH Research, GWPharmaceuticals, HMNC Brain Health, IntraCellular Therapies, Janssen, KCR, Lilly, Lundbeck, Minerva, MSD, NIH, Neumora, Novartis, Orion, Otsuka, Recognify Life Sciences, Sanofi, Servier Honoraria: Adamed, Angelini, AstraZeneca, BMS, Celon, GH Research, GSK, Janssen, KRKA, Lekam, Lundbeck, Minerva, NeuroCog, Novartis, Orion, Pfizer, Polfa Tarchomin, Sanofi, Servier, Zentiva, Consultant of: Advisory boards: Angelini, Celon (ended 2021), Douglas Pharmaceuticals, GH Research, Janssen, MSD, Novartis, Polpharma, Sanofi

## Long descriptions

### Image 1: Long description

The table contains 10 columns and 7 rows including the header. Columns are: N C T number Acronym, Number of participants, Clinical Trial Sites Locations, Age mean in years S D, Female percentage, Asian percentage, Black or African American percentage, White percentage, Ethnicity Hispanic or Latino percentage, and Ethnicity Not Hispanic or Latino percentage.

* Row 1: N C T 0 3 6 9 7 2 5 2 (completed) EMERGENT-1. 182 participants. USA (12 sites). Age 42.5 (10.11). Female 23.1%. Asian 2.2%. Black 75.3%. White 20.3%. Ethnicity N R.

* Row 2: N C T 0 4 6 5 9 1 6 1 (completed) EMERGENT-2. 252 participants. USA (22 sites). Age 45.9 (10.58). Female 24.6%. Asian 1.2%. Black 75%. White 22.6%. Hispanic 9.9%. Not Hispanic 89.3%.

* Row 3: N C T 0 4 7 3 8 1 2 3 (completed) EMERGENT-3. 252 participants. USA (20 sites) plus Ukraine (12 sites). Age 43.1 (11.82). Female 25.4%. Asian 0.4%. Black 60.9%. White 38.3%. Hispanic 12.5%. Not Hispanic 87.5%.

* Row 4: N C T 0 4 6 5 9 1 7 4 (completed) EMERGENT-4. 152 participants. USA (34 sites) plus Ukraine (10 sites). Age 44.9 (11.67). Female 25%. Asian 0.7%. Black 61.2%. White 36.8%. Hispanic 7.9%. Not Hispanic 92.1%.

* Row 5: N C T 0 5 6 4 3 1 7 0 (terminated). 4 participants. USA (5 sites). Age: Three participants between 18 and 65 years and one participant > 65 years. Female 25%. Asian 0%. Black 100%. White 0%. Hispanic 0%. Not Hispanic 100%.

* Row 6: All studies combined. 842 participants. USA (93 sites) plus Ukraine (22 sites). Female 24.7%. Asian 1.07%. Black 68.76%. White 29.45%. Hispanic 8.3% (not all studies reported data). Not Hispanic 70.43%.

Footnotes: N R = not reported; S D = standard deviation; * = not all studies reported data.

Navigate back to Image 1:.

### Image 2: Long description

The flowchart is organized into three vertical phases labeled on the left: Identification, Screening, and Included.

1. Identification Phase

- A top yellow header reads Identification of studies via databases and registers.

- A large box contains: Records identified from ClinicalTrials dot Gov (n = 5).

2. Screening Phase

- Records screened (n = 5) flows from the identification box. An arrow to the right points to Records excluded (n = 0).

- Reports sought for retrieval (n = 5) flows down from screened records. An arrow to the right points to Reports not retrieved (n = 0).

- Reports assessed for eligibility (n = 5) flows down from sought reports. An arrow to the right points to Reports excluded (n = 0).

3. Included Phase

- The final box at the bottom of the central column contains: Reports included in review (n = 5).

Navigate back to Image 2:.

